# Evaluation of clinical prediction models (part 3): calculating the sample size required for an external validation study

**DOI:** 10.1136/bmj-2023-074821

**Published:** 2024-01-22

**Authors:** Richard D Riley, Kym I E Snell, Lucinda Archer, Joie Ensor, Thomas P A Debray, Ben van Calster, Maarten van Smeden, Gary S Collins

**Affiliations:** 1 Institute of Applied Health Research, College of Medical and Dental Sciences, University of Birmingham, Birmingham B15 2TT, UK; 2 National Institute for Health and Care Research (NIHR) Birmingham Biomedical Research Centre, Birmingham, UK; 3 Julius Centre for Health Sciences and Primary Care, University Medical Centre Utrecht, Utrecht University, Utrecht, Netherlands; 4 Department of Development and Regeneration, KU Leuven, Leuven, Belgium; 5 Department of Biomedical Data Sciences, Leiden University Medical Centre, Leiden, Netherlands; 6 Centre for Statistics in Medicine, Nuffield Department of Orthopaedics, Rheumatology, and Musculoskeletal Sciences, University of Oxford, Oxford, UK

## Abstract

An external validation study evaluates the performance of a prediction model in new data, but many of these studies are too small to provide reliable answers. In the third article of their series on model evaluation, Riley and colleagues describe how to calculate the sample size required for external validation studies, and propose to avoid rules of thumb by tailoring calculations to the model and setting at hand.

External validation studies evaluate the performance of one or more prediction models (eg, developed previously using statistical, machine learning, or artificial intelligence approaches) in a different dataset to that used in the model development process.^1^ ^2^ ^3^ Part 2 in our series describes how to undertake a high quality external validation study,^4^ including the need to estimate model performance measures such as calibration (agreement between observed and predicted values), discrimination (separation between predicted values in those with and without an outcome event), overall fit (eg, percentage of variation in outcome values explained), and clinical utility (eg, net benefit of using the model to inform treatment decisions). In this third part of the series, we describe how to calculate the sample size required for such external validation studies to estimate these performance measures precisely, and we provide illustrated examples.

**Summary points**

The sample size for an external validation study should be large enough to precisely estimate the predictive performance of the model of interest
Many existing validation studies are too small, which leads to wide confidence intervals of performance estimates and potentially misleading claims about a model’s reliability or its performance compared with other models
To deal with concerns of imprecise performance estimates, rules of thumb for sample size have been proposed, such as having at least 100 events and 100 non-events
Such rules of thumb provide a starting point but are problematic, because they are not specific to either the model or the clinical setting, and precision also depends on factors other than the number of events and non-events
A more tailored approach can allow researchers to calculate the sample size required to target chosen precision (confidence interval widths) of key performance estimates, such as for R^2^, calibration curve, c statistic, and net benefit
Calculations depend on users specifying information such as the outcome proportion, expected model performance, and distribution of predicted values, which can be gauged from the original model development study
The pmvalsampsize package in Stata and R allows researchers to implement the approach with one line of code

## Rationale for sample size calculations in external validation studies

The sample size for an external validation study should be large enough to precisely estimate the predictive performance of the model of interest. The aim is to provide strong evidence about the accuracy of the model’s predictions in a particular target population, to help support decisions about the model’s usefulness (eg, for patient counselling, within clinical practice).

**Fig 1:**
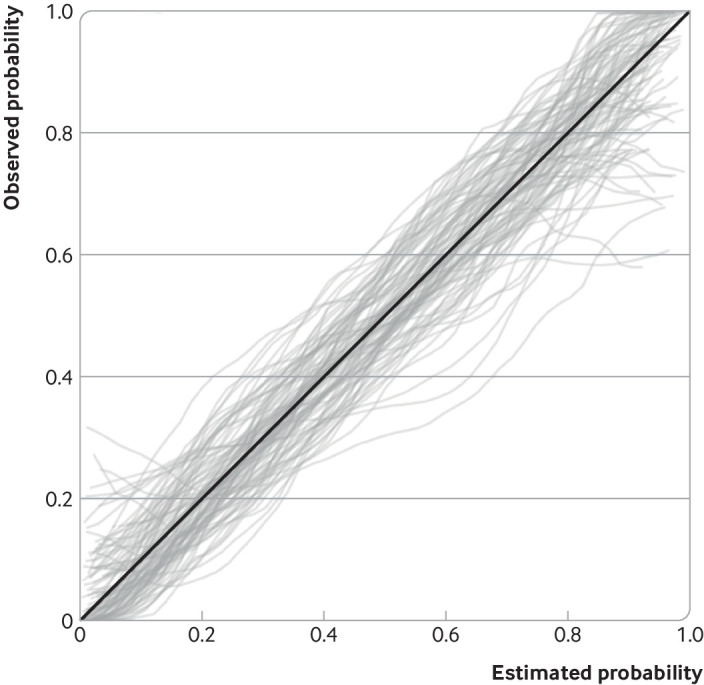
Illustration of the concern of low sample sizes when assessing calibration. Plot shows large variability in calibration curves from 100 external validation studies (each containing a random sample of 100 participants, and on average 43 outcome events, with outcomes generated assuming that the prediction model is truly well calibrated) of a prediction model for in-hospital clinical deterioration among admitted adults with covid-19

Many published external validation studies are too small, as shown by reviews of validations of statistical and machine learning based prediction models.^5^ ^6^ A small sample size leads to wide confidence intervals of performance estimates and potentially misleading claims about a model’s reliability or its performance compared with other models, especially if uncertainty is ignored.^7^ This problem is illustrated in figure 1, which shows 100 randomly generated calibration curves for external validation of a prediction model for in-hospital clinical deterioration among admitted adults with covid-19. Each curve is estimated on a random sample of 100 hypothetical participants (and about 43 outcome events), with outcomes (deterioration: yes or no) randomly generated based on assuming estimated probabilities from the covid-19 model are correct in the external validation population. Even though the model predictions are well calibrated in the population (ie, the diagonal solid line in fig 1 is the underlying truth), the sampling variability in the observed curves is large. For example, for individuals with an estimated probability between 0 and 0.05, the observed probability on the curves range between about 0 and 0.3. Similarly, for individuals with an estimated probability of 0.9, the observed probabilities on the curves range from about 0.6 to 1. Hence, the sample size of 100 participants is too small to ensure an external validation study provides stable results about calibration performance.

To resolve concerns of imprecise performance estimates, and thus inconclusive or misleading findings, rules of thumb have been proposed for the sample size required for external validation studies. For binary or time-to-event outcomes, rules of thumb based on simulation and resampling studies suggest at least 100 events and 100 non-events are needed to estimate measures such as the c statistic (area under the receiver operating characteristic curve) and calibration slope,^8^ ^9^ ^10^ and a minimum of 200 events and 200 non-events to derive calibration plots including calibration curves.^9^ ^10^ Such rules of thumb provide a starting point but are problematic, because they are not specific to either the model or the clinical setting, and precision of predictive performance estimates also depend on factors other than the number of events and non-events, such as the distribution of predicted values.^11^ ^12^ ^13^ ^14^ Therefore, rules of thumb could lead to sample sizes that are too small (producing imprecise performance estimates) or too large (eg, prospectively collecting excessive amounts of data that are unnecessarily time consuming and expensive).

To move away from rules of thumb, tailored sample size calculations are now available for external validation studies.^11^ ^12^ ^13^ ^14^ Here, we summarise these calculations for a broad audience, with technical details placed in boxes to ensure the main text focuses on key principles and worked examples. Our premise is that an external validation study aims to estimate the performance of prediction model in new data (see part 1 of our series)^15^; we do not focus on sample size required for revising or updating a prediction model. Complementary to sample size, and as emphasised in the part 2 of this series,^4^ researchers should also ensure external validation datasets are representative of the target population and setting (eg, in terms of case mix, outcome risks, measurement and timing of predictors), and typically would be from a longitudinal cohort study (for prognostic models) or a cross sectional study (for diagnostic models).

## Sample size for external validation of prediction models with a continuous outcome

**Fig 2:**
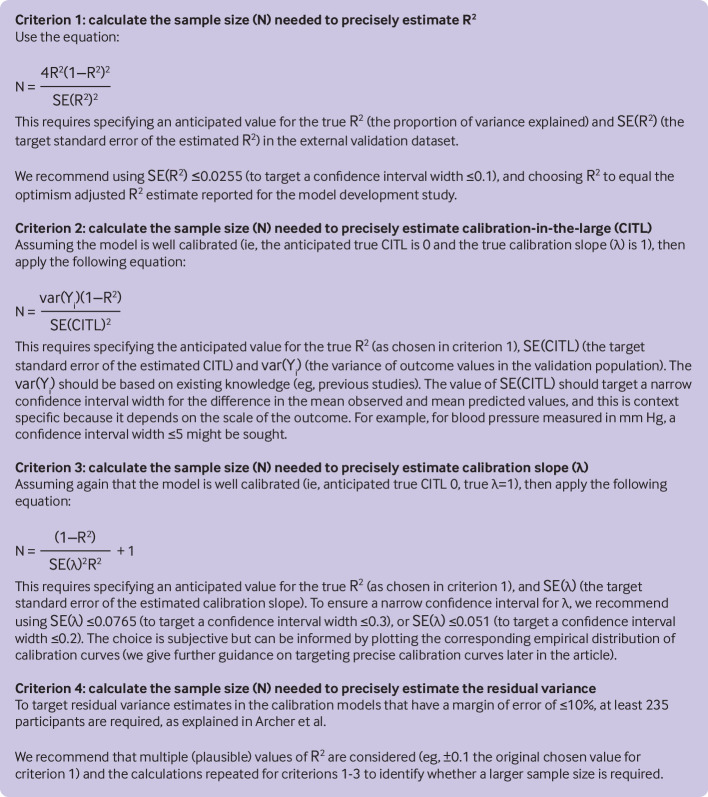
Summary of calculations for different sample sizes for external validation of a clinical prediction model for a continuous outcome (modified from Archer et al^14^), which target narrow confidence interval widths (as defined by 2×1.96×standard error) for key performance measures

When validating the performance of a prediction model for a continuous outcome (such as blood pressure, weight or pain score), there are many different performance measures of interest, as defined in detail in part 2 of this series.^4^ At a minimum, Archer et al^14^ suggest it is important to examine the following factors: overall fit as measured by R^2^ (the proportion of variance explained in the external validation dataset), calibration as measured by calibration curves and quantified using calibration-in-the-large (the difference between the mean predicted and the mean observed outcome values) and calibration slope (the agreement between predicted and observed values across the range of predicted values), and residual variance (the variance of differences between predicted and observed values in the external validation data). Four separate sample size calculations have been proposed that target precise estimation of these measures, which are summarised in figure 2. Unlike rules of thumb, the calculations are tailored to the clinical setting and model of interest because they require researchers to specify assumed true values in the external validation population for R^2^, calibration-in-the-large, calibration slope, and variance (or standard deviation) of outcome values across individuals.

Specifying these input values is analogous to other sample size calculations in medical research, for example, for randomised trials where values of the assumed effect size and target precision (or power) are needed. The dilemma is how to choose these input values. Here, we suggest that assuming values agree with those reported from the original model development study is a sensible starting point, especially if the target population (for external validation) is similar to that used in the model development study. In terms of specifying the true R^2^, we suggest use of the optimism adjusted estimate of R^2^ reported for the development study, where “optimism adjusted” refers to the estimate having been adjusted for any overfitting during development (ie, from an appropriate internal validation^15^ ^16^; see part 1 of the series^15^). In terms of calibration, we recommend that assuming the model’s predictions are well calibrated in the external validation population, such that the anticipated true calibration-in-the-large is zero and the true calibration slope is 1 (extensions assuming miscalibration are considered elsewhere^14^), corresponding to a level 2 assessment in the calibration hierarchy of Van Calster et al.^10^ The variance of outcome values can also be obtained from the model development study, or any previous studies that summarise the outcome in the target population.

Also required are the target standard errors or target confidence interval widths for the model performance estimates of interest, with the goal to ensure that 95% confidence interval widths are narrow enough to allow precise conclusions to be drawn. We assume that 95% confidence interval widths are approximated well by 2×1.96×standard error. Defining the target standard error or confidence interval width is subjective, and these values will be different for each measure (because they are on different scales), but general guidance is given in figure 2. Stata and R code to implement the entire calculation is provided at https://www.prognosisresearch.com/software, and some of the work can be implemented in the Stata and R module pmvalsampsize (see example code later). It leads to four sample sizes—one for each criterion in figure 2—and the largest of these should be taken as the minimum sample size for the external validation study, to ensure that all four criteria are met.

### Applied example: external validation of a machine learning based prediction model for pain intensity in low back pain

Lee and colleagues used individualised physical manoeuvres to exacerbate clinical pain in patients with chronic low back pain,^17^ thereby experimentally producing lower and higher pain states and recording patients’ recorded pain intensity. Using the data obtained, the researchers fitted a support vector machine to build a model to predict pain intensity (a continuous outcome ranging from 0 to 100) conditional on the values of multiple predictor variables including brain imaging and autonomic activity features. After model development, the performance was evaluated in validation data comprising 53 participants, which estimated R^2^ to be 0.40.

However, owing to the small size of the validation data, wide confidence intervals about model performance were produced (eg, 95% confidence interval for R^2^=0.20 to 0.60), and so a new external validation study is required to provide more precise estimates of performance in this particular target population. We calculated the sample size required for this external validation study using the approach outlined in figure 2, which can be implemented in the Stata module pmvalsampsize. We assumed that in the external validation population the true R^2^ is 0.40 (based on the estimate of R^2^ in the previous validation data); the model is well calibrated (ie, the anticipated true calibration-in-the-large is 0 and true calibration slope is 1); and the true standard deviation of pain intensity values is 22.30 (taken from the average standard deviation in the previous validation and training datasets in the development study). We targeted a confidence interval width ≤5 for the calibration-in-the-large (which we considered precise given the outcome scale of 0 to 100), ≤0.3 for the calibration slope and ≤0.1 for R^2^.

Applying each of the four criteria, the pmvalsampsize code is: 

pmvalsampsize, type(c) rsquared(.4) varobs(497.29) citlciwidth(5) csciwidth(.3)

This calculation suggests that the number of participants required for precise estimates is 886 for R^2^, 184 for calibration-in-the-large, 258 for calibration slope, and 235 for the residual variance. Hence, at least 886 participants are required for the external validation study, in order to target precise estimates of all four measures. If only 258 participants are recruited, then the anticipated confidence interval for R^2^ is wide (0.31 to 0.49), which would be a concern because the estimate of R^2^ not only reveals the overall model fit, but also contributes toward the estimate of the calibration slope and calibration-in-the-large (fig 2).

As the assumed R^2^ value of 0.40 is just a best guess, we repeated the sample size calculations using different values of 0.30 and 0.50. The use of 0.50 decreased the required sample size, but the use of 0.30 led to larger required sample sizes with 905 for R^2^, 214 for calibration-in-the-large, 400 for calibration slope, and 235 for the residual variance. Hence, it might be more cautious to assume an R^2^ of 0.30, and thus recruit 905 participants if possible.

## Sample size for external validation of prediction models with a binary outcome

When evaluating the performance of a prediction model for a binary outcome (such as onset of pre-eclampsia during pregnancy), researchers must examine discrimination as measured by the c statistic (ie, the area under the receiver operating characteristic curve), and calibration as measured by calibration-in-the-large (eg, the observed/expected statistic) and calibration slope.^11^ Furthermore, if the model is to be used to guide clinical decision making, then clinical utility can be measured by the net benefit statistic,^18^ ^19^ which weighs the benefits (eg, improved patient outcomes) against the harms (eg, worse patient outcomes, additional costs) of deciding on some clinical action for patients (eg, a particular treatment or monitoring strategy) if their estimated event probability exceeds a particular threshold.^18^ ^20^ These performance measures were defined in detail in part 2 of our series.^4^

**Fig 3:**
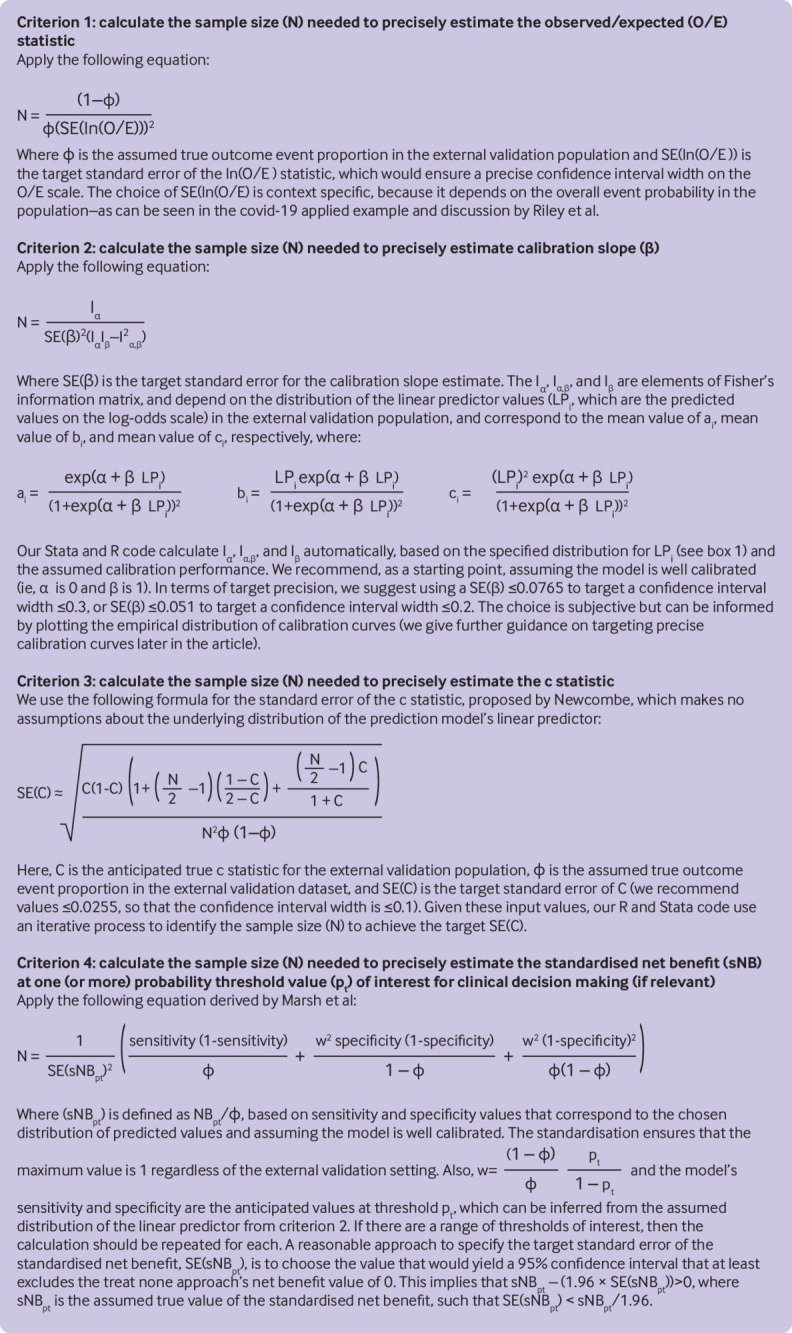
Summary of different sample size criteria for external validation of a clinical prediction model for a binary outcome, as originally proposed by Riley et al.^11^ In criterion 3, the formula for the standard error of the c statistic was proposed by Newcombe.^21^ In criterion 4, the equation applied is derived by Marsh et al^19^

To target precise estimates of these four measures of model performance, we suggest four separate sample size calculations.^11^ These calculations are summarised in figure 3 along with general guidance for choosing standard errors that target narrow confidence interval widths (defined by 2×1.96×standard error) for each performance measure.^21^ Complementary approaches have also been suggested.^19^ ^22^

As for continuous outcomes, these sample size calculations for binary outcomes also require prespecifying aspects of the (anticipated) external validation population and assumed true model performance in that population—namely, the outcome event proportion (ie, overall risk), c statistic, observed/expected statistic, calibration slope, distribution of estimated probabilities from the model, ideally specified on the log-odds scale (also known as the linear predictor distribution), and probability (risk) thresholds of interest for clinical decision making (if relevant).

Guidance for choosing these input values is given in box 1; as discussed for continuous outcomes, a sensible starting point for the performance measures is to base values on those (optimism adjusted) estimates reported from the model development study.

Box 1 **Guidance about what prespecified information is needed when applying sample size calculations for a binary outcome, modified from Riley et al^11^**

**Anticipated proportion of outcome events in the external validation population (ie, the overall risk of the outcome event)**

This proportion can be based on previous studies or datasets that report outcome prevalence (for diagnostic situations) or incidence by a particular time point (for prognostic situations) for the target population.

**Anticipated c statistic in the external validation population**

Initially, this value could be assumed equal to the optimism adjusted estimate of the c statistic reported for the model development study (or the c statistic reported for any previous validation study in the same target population), but alternative values (eg, ±0.05 of this value) can also be considered.

**Prediction model’s anticipated (mis)calibration in the external validation population**

A practical starting point is to assume that the model is well calibrated in the validation dataset, such that the anticipated true observed/expected statistic is 1 and true calibration slope is 1. Many validation studies show miscalibration, with calibration slopes less than 1 owing to overfitting in the original model development study; however, in terms of the sample size calculation, a conservative approach (as it leads to larger required numbers) is to assume a calibration slope of 1.^11^

**Distribution of the model’s estimated event probabilities in the external validation population**

This task is perhaps the most difficult, and the distribution must give the same overall outcome event proportion as assumed above. The distribution of predicted values on the log odds scale (also known as the distribution of the linear predictor or the logit transformation of the probability values) is required, and a practical starting point is to assume the same distribution as reported in the model development study. In the model development study, histograms of event probabilities are occasionally provided as part of a calibration plot, and so could be approximated by a identifying a suitable distribution on the 0 to 1 scale (eg, a beta distribution is used in our applied covid-19 example later in this article), followed by conversion to the log odds scale. Sometimes the histograms are presented stratified by outcome status, and then these can be approximated, with samples taken from each while ensuring that the overall outcome event proportion is correct.

If no direct information is available to inform the linear predictor distribution, then the assumed true c statistic can also be used to infer the distribution,^11^ ^22^ under a strong assumption that if the calibration slope is 1 then the linear predictor is normally distributed with different means but a common variance for those with and without an outcome event. We suggest that this is a last resort, because it could be a poor approximation when the assumptions break down.^11^ An alternative is to undertake a pilot study to better gauge the distribution. Such pilot data can still be included in the final sample used for external validation.

**Potential probability (risk) threshold(s) for clinical decision making (if relevant)**

These thresholds should be determined by speaking to clinical experts and patient advisory groups in the context of the decisions to be taken (eg, treatments, monitoring strategies, lifestyle changes) and the overall benefits and harms from them.

### Applied example: external validation of a model for deterioration in adults admitted to hospital with covid-19

In 2021, Gupta et al developed the ISARIC 4C deterioration model,^23^ a multivariable logistic regression model for predicting in-hospital clinical deterioration (defined as any requirement of ventilatory support or critical care, or death) among adults admitted to hospital with highly suspected or confirmed covid-19. The model was developed using data from 260 hospitals including 66 705 participants across England, Scotland, and Wales, and validated in a separate dataset of 8239 participants from London. Model performance on validation was judged satisfactory (c statistic 0.77 (95% confidence interval 0.76 to 0.78); calibration-in-the-large 0 (–0.05 to 0.05); calibration slope 0.96 (0.91 to 1.01)), and greater net benefit compared with other models. However, further external validation is now required to check that model predictions are still reliable after the introduction of covid-19 vaccines and other interventions.

**Fig 4:**
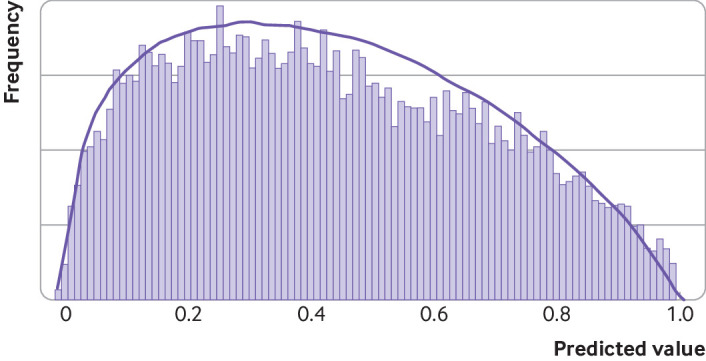
Comparison of histogram (grey bars) of predicted values (estimated event probabilities) in the validation population of Gupta et al^23^ with our assumed beta distribution (curved line) used

To calculate the sample size required, we applied the approach outlined in figure 3, with input values chosen based on box 1. We assumed that in the external validation population, the model would be well calibrated (ie, the anticipated true observed/expected statistic is 1 and true calibration slope is 1) with an anticipated c statistic of 0.77 based on the previous validation study. Further, we assumed that the distribution of the model’s event probabilities in the external validation population would be similar to that in the histogram presented by Gupta et al^23^ in their supplementary material; by trial and error, we approximated this histogram by using a beta(1.33, 1.75) distribution (fig 4), yielding a similar shape and with the same overall outcome event proportion of 0.43.

We targeted a confidence interval width of 0.22 for the observed/expected statistic (which corresponds to a small absolute error of about 0.05 compared with the assumed overall outcome event proportion of 0.43; see calculations elsewhere^11^), 0.3 for the calibration slope, 0.1 for the c statistic, and 0.2 for the standardised net benefit. Applying the sample size calculations, the corresponding Stata code is:

pmvalsampsize, type(b) prevalence(.43) cstatistic(.77)
lpbeta(1.33,1.75) oeciwidth(.12) csciwidth(.3) cstatciwidth(.1)

This calculation gives a minimum required sample size of 423 (182 events) for the observed/expected statistic, 949 (408 events) for calibration slope, 347 (149 events) for c statistic, and 38 (16 events) for standardised net benefit at a threshold of 0.1; and 407 (175 events) for standardised net benefit at a threshold of 0.3. Hence, at least 949 participants (408 events) are required for the external validation study to target precise estimates of all four measures and, in particular, to ensure calibration is properly evaluated. This sample size is much larger than the rule of thumb of 100 (or 200) events and 100 (or 200) non-events.

Additional calculations were done to see how the required sample size changed when our assumptions changed. For example, when assuming the model has the same distribution of estimated probabilities but with worse performance of either a calibration slope of 0.9 or a c statistic of 0.72, the sample size required was fewer than the 949 participants originally identified. However, if we assumed the external validation population had a narrower case mix distribution, and so used a tighter distribution of predicted values than the previous beta distribution, a sample size larger than 949 participants was required for precise estimation of the calibration slope. This change in target sample size emphasises the importance of understanding the target population and its likely distribution of predicted values. In the absence of any information, a pilot study might be useful to help gauge this distribution.

## Guidance for targeting precise calibration curves especially in regions containing thresholds relevant to clinical decision making

Calibration is often neglected in validation studies,^5^ ^24^ despite it being widely recommended, for instance as an item in the TRIPOD (Transparent Reporting of a multivariable prediction model for Individual Prognosis or Diagnosis) reporting guideline.^25^ Yet in clinical practice, predicted values (in particular, estimated event probabilities) are used for patient counselling and shared decision making—for example, to guide treatment decisions, invasive investigations, lifestyle changes, and monitoring decisions. Hence, external validation studies must produce precise estimates of calibration curves to reliably examine calibration of observed and predicted values. Ideally, curves should be precise across the whole range of predicted values, which is why our sample size criteria aims to estimate calibration-in-the-large (observed/expected statistic) and calibration slope precisely. At the bare minimum, curves should be precise within regions containing possible probability thresholds relevant for clinical decision making. We discuss this topic further in the supplementary material.

Very large sample sizes might be needed to estimate the whole calibration curve precisely. Furthermore, choosing the target standard errors for the calibration measures (slope and calibration-in-the-large) is subjective and difficult to gauge, especially for binary outcomes because the slope is estimated on the logit scale. To deal with this problem, we suggest plotting the empirical distribution of calibration curves that arise from a dataset with the sample size identified based on particular chosen target standard errors (eg, corresponding to a confidence interval width of 0.3 for the calibration slope), to check whether their variability is sufficiently low, especially in regions encompassing thresholds relevant to decision making. This approach can be done as follows:

- Simulate a large number of datasets (eg, 100 or 200) with the sample size identified (for the chosen target standard errors), under the same assumptions as used in the sample size calculation (eg, assumed calibration slope of 1, same distribution of predicted values).
- For each dataset separately, derive a calibration plot including a calibration curve, as described in the second paper in this series.^4^
- On a single plot, overlay all the calibration curves to reveal the potential range of calibration curves that might be observed in practice for a single external validation study of that sample size. If variability is considered to be high on visual inspection, then a larger sample size is needed. Conversely, if variability is very precise, a lower sample size might suffice, especially if the original sample size is not considered attainable.
- As mentioned, at the bare minimum, researchers should ensure low variability of curves in regions of most relevance for clinical decision making.

**Fig 5:**
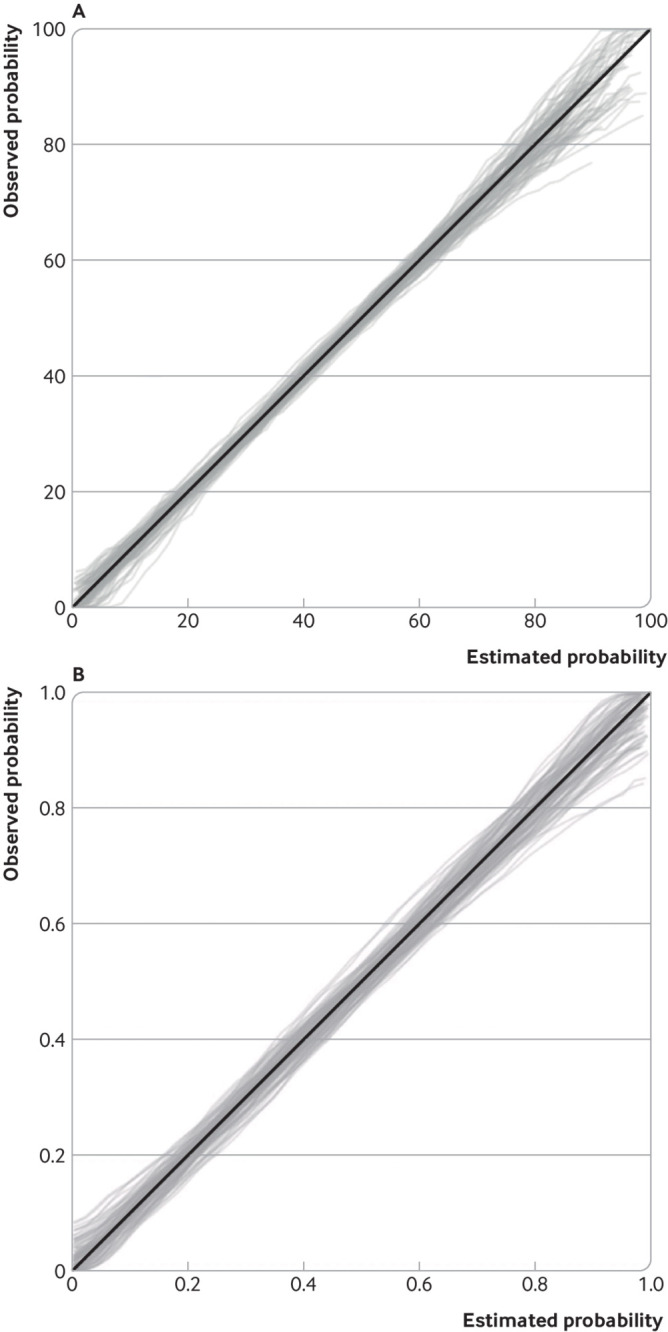
Distribution of calibration curves for (A) pain intensity prediction model (based on 258 participants) and (B) covid-19 deterioration prediction model (based on 949 participants (408 events)), derived from 100 simulated datasets with the sample size required to estimate the calibration slope precisely according to a target confidence interval width of 0.3 (standard error ≤0.0765) for the calibration slope. Simulations assume that the models are well calibrated, with a true calibration slope of 1 and calibration-in-the-large of zero

To illustrate this approach, figure 5 shows the empirical distribution of 100 calibration curves for our two applied examples when simulated using the sample size previously calculated to target a standard error of 0.0765 (confidence interval width of 0.3) for the calibration slope. For the pain intensity continuous outcome, 258 participants were identified as necessary for estimating calibration slope assuming it was 1, and the corresponding distribution of curves based on this sample size is reasonably narrow (fig 5A). At predicted values less than 80, the spread of observed curves corresponds to a difference in pain score of only about 5 to 10. Only at the upper end is the variability more pronounced (differences up to about 20), for example, with curves spanning pain scores of 80 to 100 at a predicted score of 90. If values in this range represent thresholds critical to clinical decision making, then larger sample sizes might be required. However, any value over 80 is likely to be always classed as very high, and so the observed variability in this upper range is unlikely to be important. Hence, the calculated target sample size of 258 participants still seems sensible.

For the covid-19 deterioration model with 949 participants (408 events), the variability is also quite narrow across the entire range of event probabilities, although slightly larger at very high probabilities (0.8 to 1); the spread of observed curves corresponds to a difference in observed probabilities of about 0.05 to 0.15 in most regions, which is reasonably precise. If the target confidence interval width for the calibration slope is narrowed to 0.2 (rather than 0.3), then the minimum required sample size increases dramatically to 2137 participants (918 events), and yet the reduction in variability of the calibration curves is relatively small (supplementary fig S1), with differences in observed probabilities of about 0.05 to 0.10 in most regions. Hence, such a large increase in sample size (with costs and time in participant recruitment, for example) might be difficult to justify.

Conversely, the 100 or 200 events rule-of-thumb corresponds to 233 or 466 participants, respectively, which leads to a wider variability of observed calibration curves spanning a difference in observed probabilities of about 0.15 to 0.2 (200 events) to 0.2 to 0.25 (100 events) in most regions (supplementary fig S2), and introduces much more uncertainty about calibration agreement, including in ranges where high risk thresholds (eg, between 0.05 and 0.1) might exist. Hence, reducing the target sample size also does not appear justified here, and so the originally calculated 949 participants (408 events) still reflects a sensible and pragmatic target for the external validation study in terms of calibration.

## Extensions

### Missing data in external validation of continuous or binary outcome prediction models

So far, we assumed that the external validation study had no missing data, but in practice some participants could have missing outcomes (eg, due to loss to follow-up) or missing predictions (eg, due to missing values of predictors in the model). In such situations, inflating the original sample size to account for potential missing information is helpful. For example, if 5% of participants are anticipated to have missing outcomes or predictor values, then 999 participants should be recruited (999×0.95=949, the sample size calculated earlier based on complete data).

### Prediction models with time-to-event outcomes

Extension to external validation of prediction models with a time-to-event (survival) outcome is challenging, because closed form (ie, analytical) calculations are difficult to derive. To resolve this problem, we suggest a simulation based approach to assess the precision of estimates of calibration, discrimination, and net benefit.^12^ ^13^ In brief, external validation datasets of a particular sample size are simulated under assumptions about the event and censoring distributions, the length of follow-up, the model’s linear predictor distribution, and the (mis)calibration performance. Then, for each external validation dataset, predictive performance and calibration curves are estimated at each time point, and the extent of their precision and variability examined. Stata and R code are available at https://www.prognosisresearch.com/software.^12^ Jinks et al consider sample size for precise estimation of Royston’s D statistic.^26^

### Planning to obtain existing datasets

Many external validation studies plan to obtain an existing dataset with a fixed sample size (rather than recruit new participants). In that situation, our approaches can be adapted to calculate the expected precision (confidence interval width) conditional on that dataset’s sample size and any other known characteristics (eg, distribution of estimated probabilities, observed variance of continuous outcomes, observed outcome event proportion, censoring rate). This calculation will help researchers and potential funders to ascertain whether the dataset is large enough (eg, to justify any costs and time involved in obtaining the data), while also considering other quality aspects (eg, setting, recording of predictors, measurement methods). Sample sizes can be increased further by combining data from multiple sources, such as individual participant data from multiple studies or electronic healthcare records across multiple practices or hospitals.^27^ ^28^

### Clear and transparent reporting

With regard to sample size, the TRIPOD reporting guideline prompts authors to explain how the study size was arrived at.^16^ ^25^ In the context of our proposed sample size calculations for external validation studies, for a continuous outcome (fig 3) this reporting item would entail providing the anticipated values for R^2^, calibration-in-the-large, and calibration slope together with target standard errors or confidence interval widths. For a binary outcome (box 1), this reporting item would entail providing the assumed outcome event proportion, observed/expected statistic, calibration slope, distribution of the linear predictor (eg, beta distribution and its parameters), and c statistic, together with target standard errors or confidence interval widths, and any probability thresholds (for clinical decision making).

## Conclusions

This article concludes our three part series on evaluation of clinical prediction models, in which we have discussed the principles of different types of evaluation,^15^ the design and analysis of external validation studies,^4^ and, here, the importance of sample size calculations to target precise assessments of a prediction model’s performance. This article complements related work on sample size calculations for model development.^29^ ^30^ ^31^ ^32^

## Web extra

Extra material supplied by authors

Web appendix: Supplementary material

## Notes

Contributors: RDR, KIES, LA, and GSC led the previous methodology papers that underpin the sample size proposals in this article, with contributions for improvement made at various stages by JE, TPAD, MvS, and BVC. RDR wrote the first and updated drafts of this article, with subsequent contributions and revisions by GSC, then by all authors at multiple stages. RDR, GSC, and JE wrote the corresponding code to implement the methods in Stata and R. JE led the pmsampize package development. RDR is the guarantor. The corresponding author attests that all listed authors meet authorship criteria and that no others meeting the criteria have been omitted.

Patient and public involvement: Patients or the public were not involved in the design, or conduct, or reporting, or dissemination of our research.

Provenance and peer review: Not commissioned; externally peer reviewed.
