# Supplementary material for: Evaluation of clinical prediction models (part 3): calculating the sample size required for an external validation study
Source: BMJ. 2024 Jan 22;384:e074821. doi: 10.1136/bmj-2023-074821 (PMC11778934; doi:10.1136/bmj-2023-074821)
Supplement: Supplementary file 1 — Web appendix: Supplementary material [file rilr074821.ww.pdf]

Supplementary material

Figure S1. Distribution of calibration curves for (a) the pain intensity prediction model and (b) the COVID-19 deterioration prediction model derived from 100 simulated datasets with the sample sizes shown aiming to target a confidence interval width of 0.2 for the calibration slope The simulations assume the models are well calibrated, with a true calibration slope of 1 and calibration-in-the-large of zero.

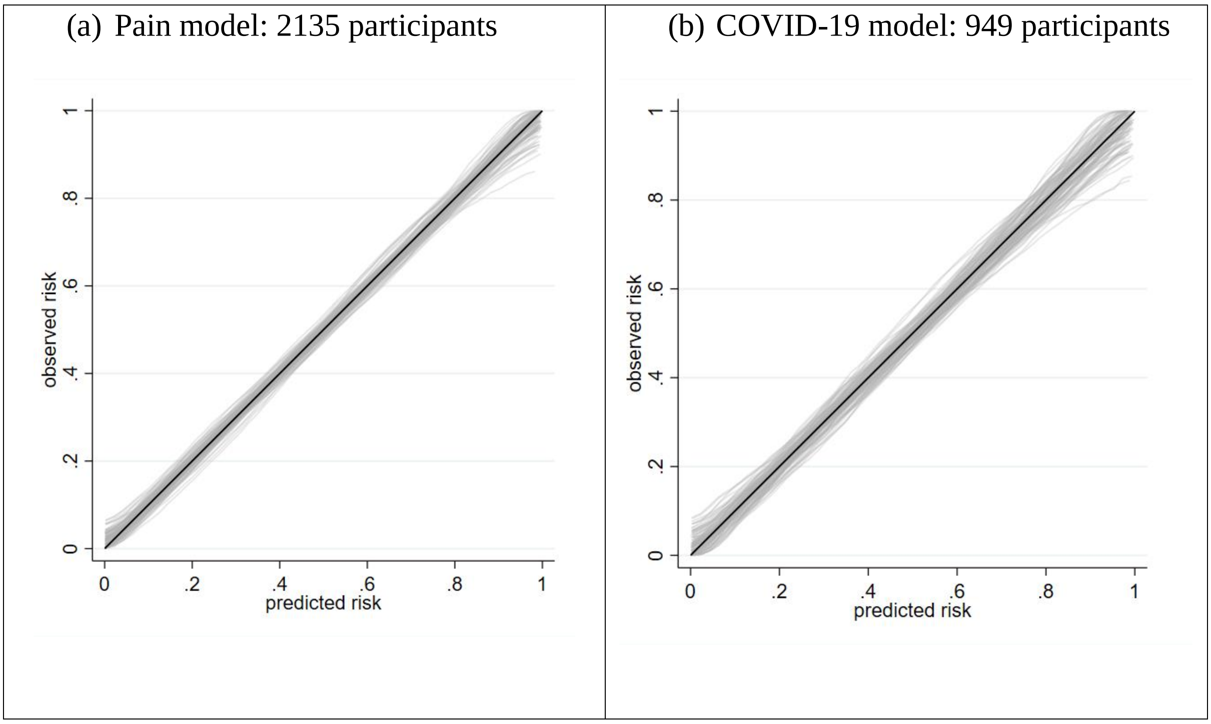

Figure S2. Distribution of calibration curves for the COVID-19 deterioration prediction model derived from 100 simulated datasets with the sample size required to estimate the calibration slope precisely according to about (a) 200 events or (b) 100 events. The simulations assume the models are well calibrated, with a true calibration slope of 1 and calibration-in-the-large of zero.

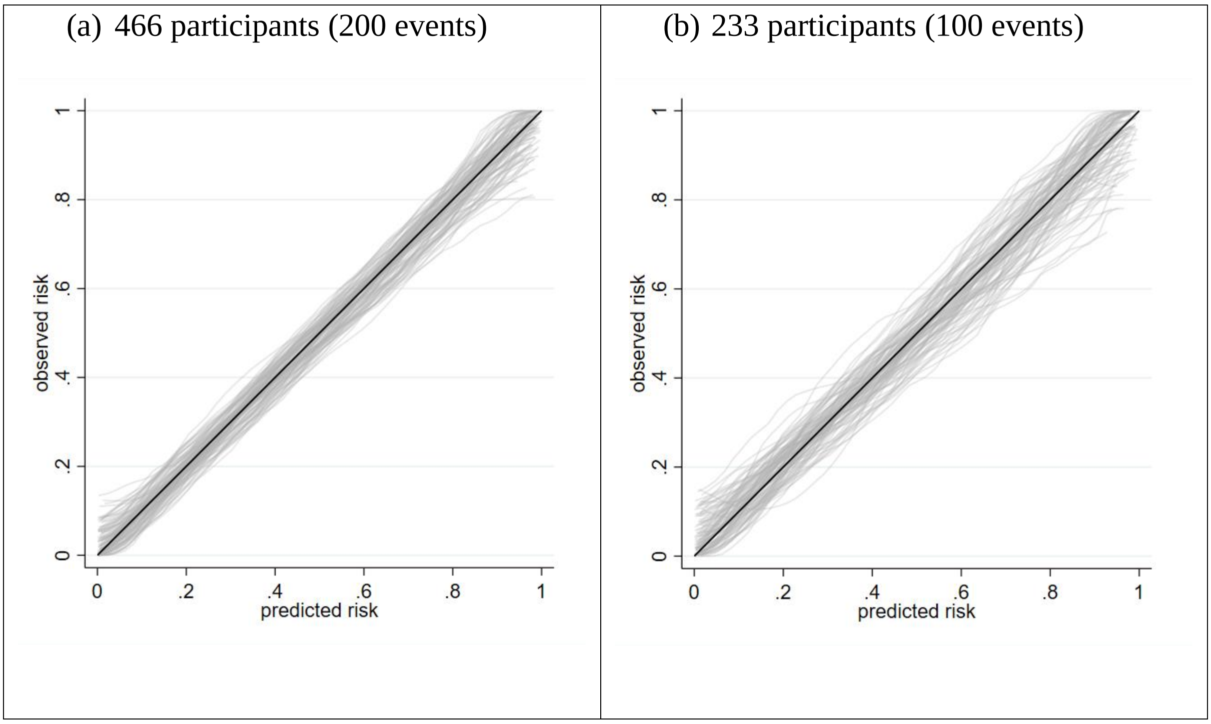

Should we only focus on precise calibration in sub-regions of predicted values?

Ideally, sample size calculations for calibration should aim for a precise calibration curve across the entire range of predictions. An alternative (narrower) view is to ensure sample size is large enough to precisely estimate calibration in regions of predicted values relevant to clinical decision making, but to be less concerned about imprecision in other regions. For example, sometimes a probability threshold is used to define when risks are high enough to warrant some clinical action (e.g., 10%), and so precision of the calibration curve is most important in the region close to this threshold (e.g., 0 to 20%), and potentially less important in regions further away (e.g., 80 to 100%). In this special situation, lower sample sizes for calibration may be acceptable than when aiming for precise curves across the whole range. This should only be considered if feedback from advisory groups (including patient representatives, clinicians and other healthcare decision makers) is clear about a strict range of thresholds of interest,[26] and should not be driven by the desire to lower the sample size itself.
